# Public attitudes towards the use of novel technologies in their future healthcare: a UK survey

**DOI:** 10.1186/s12911-023-02118-2

**Published:** 2023-02-22

**Authors:** Sarah Sauchelli, Tim Pickles, Alexandra Voinescu, Heungjae Choi, Ben Sherlock, Jingjing Zhang, Steffi Colyer, Sabrina Grant, Sethu Sundari, Gemma Lasseter

**Affiliations:** 1 National Institute for Health Research Bristol Biomedical Research Centre, University Hospitals of Bristol and Weston NHS Foundation Trust and University of Bristol, Bristol, UK; 2 Centre for Trials Research, College of Biomedical and Life Sciences, Cardiff University, Cardiff, UK; 3 Department of Psychology, University of Bath, Bath, UK; 4 School of Engineering, Cardiff University, Cardiff, UK; 5 College of Medicine and Health, University of Exeter, Exeter, UK; 6 Department of Mathematical Sciences, University of Essex, Colchester, UK; 7 Department of Health, University of Bath, Bath, UK; 8 Musculoskeletal Research Unit, Translational Health Sciences, Bristol Medical School, University of Bristol, Bristol, UK; 9 School of Nursing and Midwifery, University of Worcester, Worcester, UK; 10 NIHR Health Protection Research Unit (HPRU) in Behavioural Science and Evaluation, University of Bristol in Collaboration with UK Health Security Agency (UKHSA), Bristol Medical School, Population Health Sciences, University of Bristol, Bristol, UK

**Keywords:** Novel healthcare technologies, Survey, Acceptability, Attitudes, Laser, Microwave signals, Virtual reality, Mobile application

## Abstract

**Background:**

Innovation in healthcare technologies can result in more convenient and effective treatment that is less costly, but a persistent challenge to widespread adoption in health and social care is end user acceptability. The purpose of this study was to capture UK public opinions and attitudes to novel healthcare technologies (NHTs), and to better understand the factors that contribute to acceptance and future use.

**Methods:**

An online survey was distributed to the UK public between April and May 2020. Respondents received brief information about four novel healthcare technologies (NHTs) in development: a laser-based tool for early diagnosis of osteoarthritis, a virtual reality tool to support diabetes self-management, a non-invasive continuous glucose monitor using microwave signals, a mobile app for patient reported monitoring of rheumatoid arthritis. They were queried on their general familiarity and attitudes to technology, and their willingness to accept each NHT in their future care. Responses were analysed using summary statistics and content analysis.

**Results:**

Knowledge about NHTs was diverse, with respondents being more aware about the health applications of mobile apps (66%), followed by laser-based technology (63.8%), microwave signalling (28%), and virtual reality (18.3%). Increasing age and the presence of a self-reported medical condition favoured acceptability for some NHTs, whereas self-reported understanding of how the NHT works resulted in elevated acceptance scores across all NHTs presented. Common contributors to hesitancy were safety and risks from use. Respondents wanted more information and evidence to help inform their decisions, ideally provided verbally by a general practitioner or health professional. Other concerns, such as privacy, were NHT-specific but equally important in decision-making.

**Conclusions:**

Early insight into the knowledge and preconceptions of the public about NHTs in development can assist their design and prospectively mitigate obstacles to acceptance and adoption.

**Supplementary Information:**

The online version contains supplementary material available at 10.1186/s12911-023-02118-2.

## Background

In the United Kingdom there are approximately 12 million older people (age 65+), of which 40% live with a longstanding, limiting disease [1]. An increase in physical disability and chronic disease among younger adults is expected to raise future demand for social care and disability benefits [2]. Furthermore, the annual National Health Service (NHS) costs attributable to excess weight, a modifiable risk factor for multiple noncommunicable diseases, are projected to reach £9.7 billion by 2050 [3]. The economic burden of disease prevention and treatment has been further amplified by the COVID-19 pandemic [4, 5].

To mitigate the rising costs of health and social care, in 2011 the Department of Health released a new strategy that placed technological innovation at the forefront of policy making [6]. The strategy’s objective was to harness the potential of technology to propel improvements in quality and efficiency [7]. Areas where technological innovation proved to be particularly valuable include enabling early diagnosis [8]), empowering patients to gain control and choice in their treatment [9], and facilitating remote monitoring for timely intervention [10]).

Despite the potential advantages of technological innovation in health and care, uptake in routine clinical practice has been generally poor. From 2009 to 2014, only 34% of technologies presented to the UK’s National Institute for Health and Care Excellence were recommended for NHS adoption [11]. Adoption and diffusion of approved technologies is often slow, disrupted, and inequitable [12, 13]. For example, videoconferencing for remote consultations and monitoring is not new, but nation-wide attempts to its implementation prior to the COVID-19 pandemic have had limited success [14].

A key determinant of the successful implementation of innovative healthcare technology is the attitudes of health professionals and service users [15], as these play an important role in guiding behaviour [16]. There is no clear definition of ‘innovation’ in healthcare technology, which can be characterized as any degree of modification to a technology already in use in the attempts to improve treatment, the adaptation of an existing technology for a novel therapeutic area, or the development of a healthcare solution stemming from unprecedented combinations of technological components. This is accompanied by the relatively less established regulatory scrutiny over innovation in healthcare technology in comparison to pharmaceutical innovation at a global level. Hence, the development, evaluation, deployment and adoption of healthcare technologies is vulnerable to attitudinal biases of individual agents involved in these processes [17].

Considering that the average lag time for technological innovation to be translated into routine practice is 17 years [18] and that this process requires significant investment, there is extensive value in obtaining an early understanding of attitudes towards a technology in development. It can guide process evaluations and the development of implementation strategies that focus on what is important to stakeholders. It can also contribute to establishing policies that overcome ethical issues identified when innovation is introduced in clinical settings, such as reducing conflicts of interest and obtaining informed consent [17, 19].

As demonstrated by research on digital health interventions [20, 21], attitudes towards healthcare technology is highly variant between different groups of patients and the public, and it is heavily influenced by knowledge about the technology, perceived quality of the technology, and how the technology is endorsed. For example, uptake of tracking apps during COVID-19 was poor, despite the widespread use of mobile apps that normally monitor the user’s location, and this phenomenon was partially attributable to public distrust [22]. Existing physical activity interventions utilising digital technology have been found to be much less effective in low compared to middle-high socioeconomic groups [23]. While attitudinal barriers have often been associated with the low uptake of technology by older age groups, these have been found to be modifiable by education and experience [24].

As researchers strive to create innovative technologies that enable targeted and timely interventions, the present study aimed to capture UK public opinions and attitudes to a range of novel healthcare technologies (NHTs). The vast majority of research in this area has focused on evaluating a singular technological solution, the comparison of similar technologies (e.g., digital health) or a range of technologies in the same therapeutic area (e.g., endocrinology). In doing so, however, we run the risk of using the existing evidence base to make incorrect assumptions on how population groups may respond to future innovation and factors influencing value-judgments made by patients when considering adoption of NHTs.

The specific objectives of this study therefore were: (i) evaluate the public’s acceptability of a range of NHTs in their future care; (ii) discern the breadth of diversity in self-reported predispositions towards these technologies; and (iii) seek initial insight into how these technologies could be introduced to patients in the future. Unlike previous research, we presented four different technologies in development, and asked respondents the same set of questions. By facilitating comparability between technologies, this study attempted to explore whether knowledge, attitudes and beliefs towards the introduction of new technology can be generalised to all NHTs or is contingent on the technology and its proposed application.

## Methods

### Study design and setting

A cross-sectional online survey.

### Population, sampling, and data collection

The survey was distributed online and managed by PureProfile, a professional social research company with over 140,000 registered individuals with a range of characteristics representative of the UK population geographically and demographically. Members of the public registered with PureProfile were eligible to participate if they were aged 18 years or above and lived in the UK. The survey was open for recruitment from the 30 April 2020 to 20 May 2020.

### Ethical approval

Ethical approval was obtained from the Faculty of Health Sciences Research Ethics Committee, University of Bristol (Ref: 94502).

### Survey development

Preliminary interviews were conducted by co-authors (HC, SG, BS, TP, SS, AV) to define ‘NHT' with 12 healthcare professionals (HCPs) who had diverse expertise, seniority, degree of patient contact. These interviews further explored HCPs views and experiences of implementing NHTs in the UK national healthcare system, barriers and facilitators. There was consensus among the HCPs that the possible definitions of NHTs were manyfold; all new technologies implemented outside of standard practice can be defined as NHTs if they aim to improve health and social care provision in the form of new medicine, devices, procedures, and/or techniques. Grounded on insight from the interviews, ‘novelty’ could be attributed to the actual technology underpinning a medical device or the health application of an existing technology.

Finding from these interviews were used to draft the survey by all co-authors; an interdisciplinary team with expertise across the translational pathway of health technology development and implementation evaluation. To minimise the risk that differential interpretation of ‘NHT’ would confound survey responses, four NHTs targeting well known and common chronic conditions were used in the survey as case studies. All were either being researched or in the commercial development phase at the time the survey was distributed. Table 1 displays technologies selected and differences/overlaps between them (see Additional file 1 for details on the case studies).

**Table 1:** Characteristics of NHTs included in the survey

	NHT ‘type’	Function	Technology	Medical condition	Anticipated resistance to acceptance prior to the survey
Laser-based tool for early diagnosis	Completely new	Early diagnosis	Nonlinear microscopy	Osteoarthritis (1 in 10 adults in UK;[25])	No cure exists for this condition
Virtual reality to support self-management	Novel application, existing technology	Treatment	Virtual reality	Diabetes Mellitus/Obesity (7% of UK [26]/28% of adults in England[27])	Public ‘anxiety’ around gaming Ownership of data Expectations of dizziness and tiredness from use
Continuous glucose monitoring using microwave signals (Heungjae, 2015)	Completely new	Self-monitoring	Microwaves	Diabetes Mellitus (7% of UK population [26])	Public ‘anxiety’ around microwave exposure Myth of microwaves causing cancer Relational memory linked to using domestic microwave ovens
Mobile app for patient reported monitoring	Novel application, existing technology	Self-monitoring	Smartphone application	Rheumatoid arthritis (1% of UK population [28]	Ownership of data Privacy

The structure of the survey comprised both multiple-choice and open questions across the following sections (see Additional file 1):

- Familiarity with technology: Questions targeted overall technology use as well as use of technologies to manage own health.
- Presentation of case studies of the NHTs: First, survey items captured preliminary knowledge and predispositions towards the technology. Second, respondents were asked to indicate (on a Likert Scale) how likely they would agree to the use of the NHT after reading (a) information about the NHT and its proposed application, (b) the benefits, (c) the risks. Three separate agreement scores were provided for each NHT, ranging between 1 (“Strongly Agree”) and 5 (“Strongly Disagree”). Finally, survey items captured respondent’s views on who and how the NHTs should be introduced to them.
- Experience with monitoring technology: Data were captured on use of this technology, opinions regarding access to information collected by the technology, and comfort in sharing sensitive information via the technology.
- Sociodemographic characteristics: Age, gender, ethnicity, index of maximal deprivation, education, general health.

Prior to launching the survey, an NIHR Bristol Biomedical Research Centre Patient and Public Involvement group piloted the survey and provided feedback. Two group sessions were run. The information was collated and incorporated in subsequent iterations of the survey.

The final draft of the survey was digitalised by the PureProfile team and piloted on 100 participants via their online platform. Modifications were made to the layout, format and logic of the questions prior to launch. The survey was distributed randomly by PureProfile, but quotes were applied for age and gender to ensure responders were a nationally representative sample. Respondents were provided with £2.10 payment by PureProfile for the time taken to complete the survey. Anonymised survey data were provided to the study team for analysis and the final sample size included the pilot responses.

### Statistical analysis

Summary statistics show participant responses to survey questions. For case study responses, a single agreement score was calculated by averaging the three agreement scores provided. The lower the score, the higher the degree of agreement to accepting the NHT in future care. All three scores were required to calculate the average, otherwise the agreement score was left missing for the participant. Index of Multiple Deprivation (IMD) Quintile was determined from responders’ postcodes provided in the survey (see Additional file 2 for details).

Two open-ended questions were included in the survey to explore respondents’ reluctance to accept the NHTs presented in the case studies and views on how health professionals should introduce the presented NHTs to patients in the future. These open-ended free text questions were analysed using content analysis. The first 50 responses to the two questions were coded independently by the research team (SS, AV, HC, TP for question 1; SS, SeS, HC, GL for question 2). A coding framework was developed, after which researchers coded the remaining responses. SS further coded 10% of all responses to check for consistency. The frequency and percentage of codes that emerged in the responses were calculated.

Data were provided from PureProfile in IBM SPSS Statistics 25 and all descriptive analyses were undertaken in this package.

## Results

Preliminary interviews with HCPs revealed that the survey would have to provide participants with a definition of ‘Novel Healthcare Technology’ and that the technology should be presented alongside its proposed application (i.e., the medical condition). HCPs highlighted that at present there is a lack of standard procedure through which patients are introduced to NHTs and there is a need to identify and adequately train the key people involved in this process. This information was used to shape the survey and incorporate additional questions around respondents’ views on how NHTs should be introduced to future patients.

### Sample

A total of 1450 adults responded to the survey. Median time taken to complete the survey was 15.60 min (range 4.77–1958.5 min). Respondents were based across the United Kingdom, though most were from London (13.2%) and the South East England (13.9%).

Mean age of participants was 46.4 years (SD: 17.13) and 50.6% were female. The majority of respondents were British White (80.1%). 6.3% were Irish or Other white background, 3.1% were Asian, 2.7% were mixed race, 1.8% were black and 1.2% were Chinese. The overall distribution of the sample aligned with population data on ethnicity collected by the Office of National Statistics [29]. Most respondents were in active paid work (56.4%), 32.1% held a University degree, and IMD score was evenly spread across quantiles. Respondents mainly rated their general health as ‘Good’ (57.2%), and only 3.5% indicated ‘Bad’ or ‘Very Bad’. 63.9% of participants indicated they had no health condition, with most participants who reported a health condition classifying it as ‘long-term illness, disease or condition’ (14.4%) (see Additional file 2 for details).

### Knowledge and use of NHTs

Overall, a large majority of participants indicated they were frequent users of technology in their everyday life (82.6%), though only 9.4% reported that novel technologies were being used for the management of their health. Of respondents who had previously used NHTs (n = 225), most made use of these technologies every few months (25.3%) or once every few years (27.1%).

**Fig. 1:**
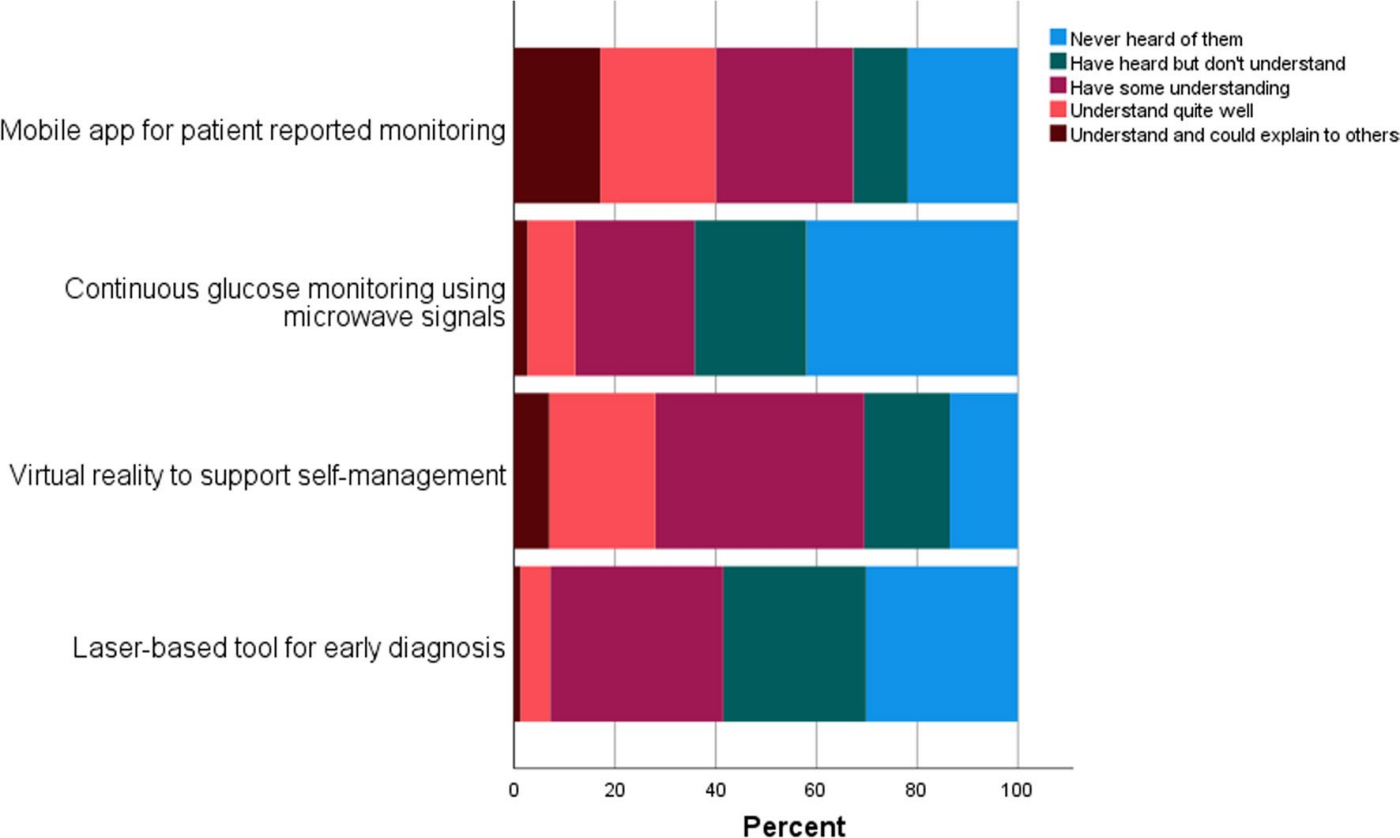
Degree of respondents’ understanding of the NHTs presented (n = 1450)

**Fig. 2:**
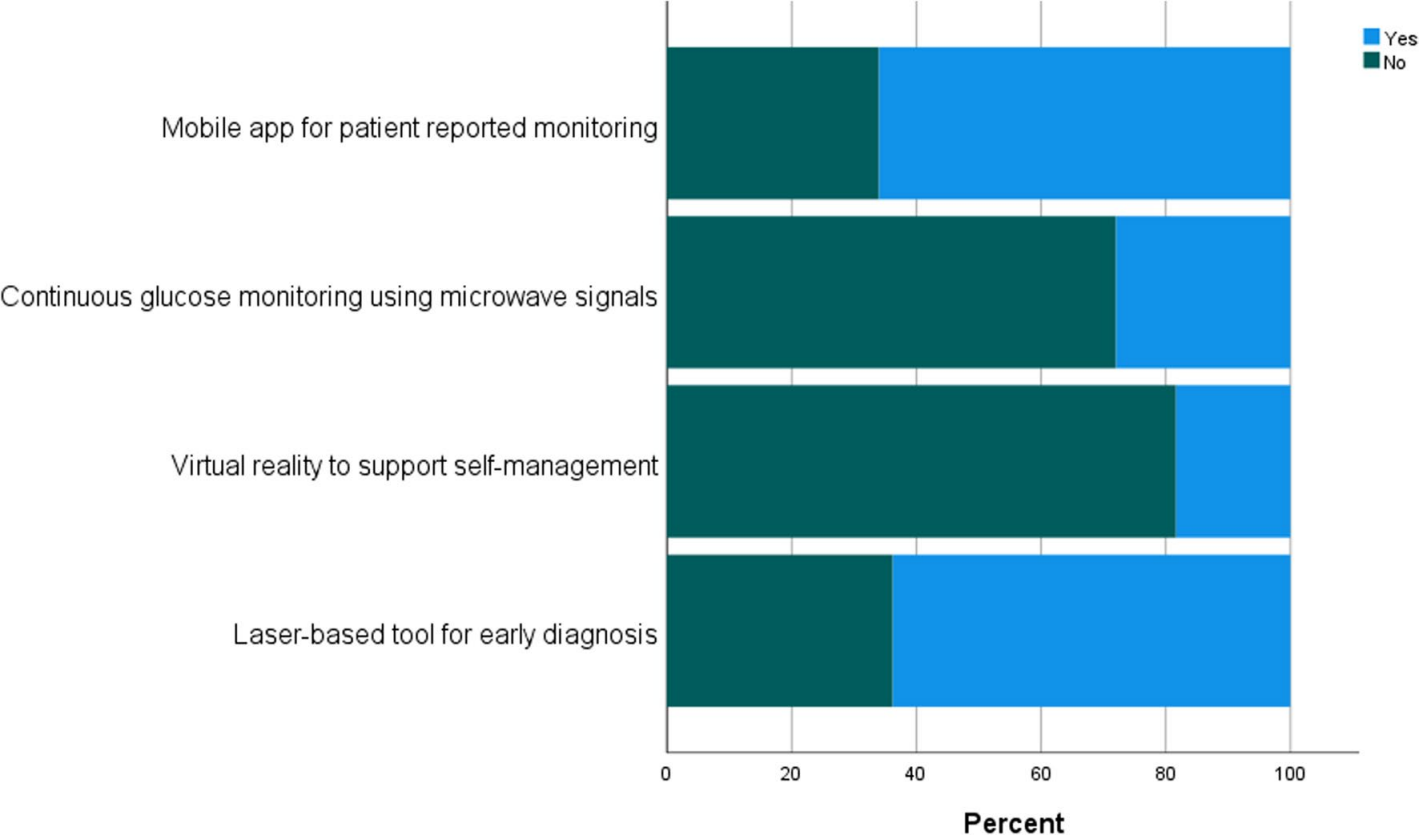
Percentage respondents who were the technologies presented were being used in healthcare. (n = 1450)

Regarding the NHT case studies included in the survey, Fig. 1 displays participants’ previous understanding of how the actual technology works. Self-reported knowledge on laser-based and microwave-based technology primarily ranged between ‘some understanding’ to ‘never heard of them’, while responses were more diverse for mobile app and virtual reality technology. Figure 2 presents whether respondents were aware of the use of these technologies in healthcare. Awareness was highest for mobile apps (66%), followed by laser-based technology (63.8%), microwave technology (28%) and lastly virtual reality (18.3%).

### Acceptability of NHTs in future care

After reading information about the NHTs and the proposed health application, including associated benefits and risks, median acceptance score provided by respondents was 2.0 for virtual reality to support self-management (Interquartile Range (IQR): 1.3–3.0) and continuous glucose monitoring using microwave signals (IQR: 1.3–2.3), and 1.7 for laser-based technology for early diagnosis (IQR: 1.3–2.0) and mobile app for patient reported monitoring (IQR: 1.0–2.0). Agreement scores ranged between 1 “Strongly agree” to 5 “Strongly disagree”.

### Acceptability according to individual characteristics

**Fig. 3:**
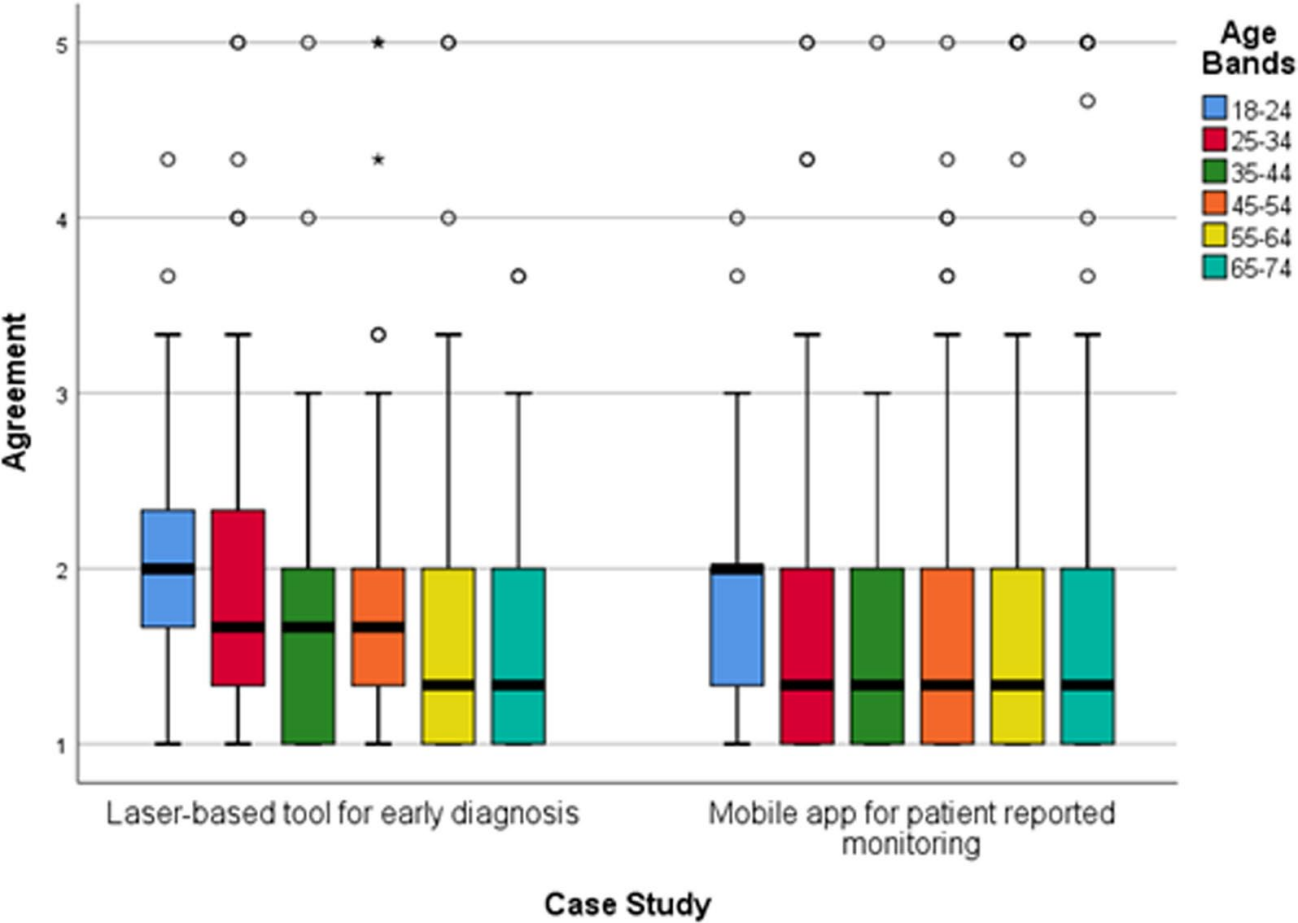
Degree of agreement to accept the laser-based technology and mobile apps in future care according to respondents’ age. Agreement score ranges between 1 “Strongly agree” to 5 “Strongly disagree”. Circles indicate mild outliers (between 1.5 and 3 interquartile ranges away from the 75th percentile) and stars indicate extreme outliers (greater than 3 interquartile ranges away from the 75th percentile). Laser-based tool for early diagnosis n = 1139. Mobile app for patient reported monitoring n = 1093

**Fig. 4:**
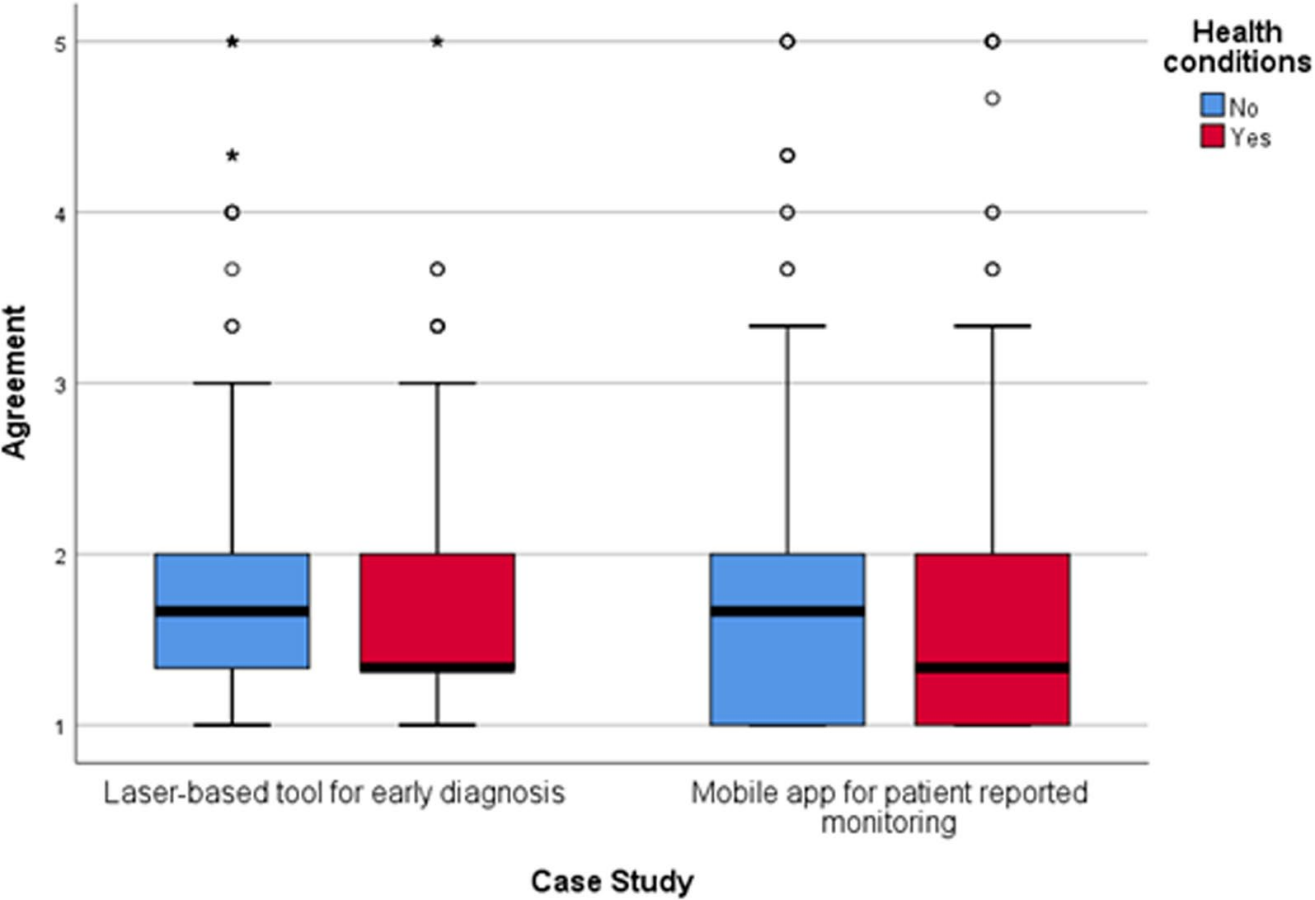
Degree of agreement to accept laser-based technology and mobile apps in future care, split by respondents who reported having a medical condition and those who didn’t. Agreement score ranges between 1 “Strongly agree” to 5 “Strongly disagree”. Circles indicate mild outliers (between 1.5 and 3 interquartile ranges away from the 75th percentile) and stars indicate extreme outliers (greater than 3 interquartile ranges away from the 75th percentile). Laser-based tool for early diagnosis n = 1139. Mobile app for patient reported monitoring n = 1093

Using cross tabulation, several patterns were detected in acceptance according to respondent characteristics. Increasing age was linked with an increased willingness to adopt laser-based technology for early diagnosis and a mobile app for patient reported monitoring (Fig. 3). Age had no effect on acceptance scores for the other NHTs. Presence of a self-reported condition followed a similar pattern, having a positive effect on acceptance in the case studies depicting laser-based technology and mobile apps, but not the other NHTs (Fig. 4). A gender difference was only found in acceptance of mobile apps for patient reported monitoring, being slightly elevated in females (median: 1.3; IQR: 1.0–2.0) compared to males (median: 1.7; IQR: 1.0–2.0). Agreement ratings towards the mobile apps were also closer to “Strongly agree” for those aware of the use of these apps in healthcare (median: 1.3; IQR: 1.0–2.0) compared to those not aware (median: 2.0; IQR: 1.0–2.3). These patterns were not observed for the other NHTs (see Additional file 3).

**Fig. 5:**
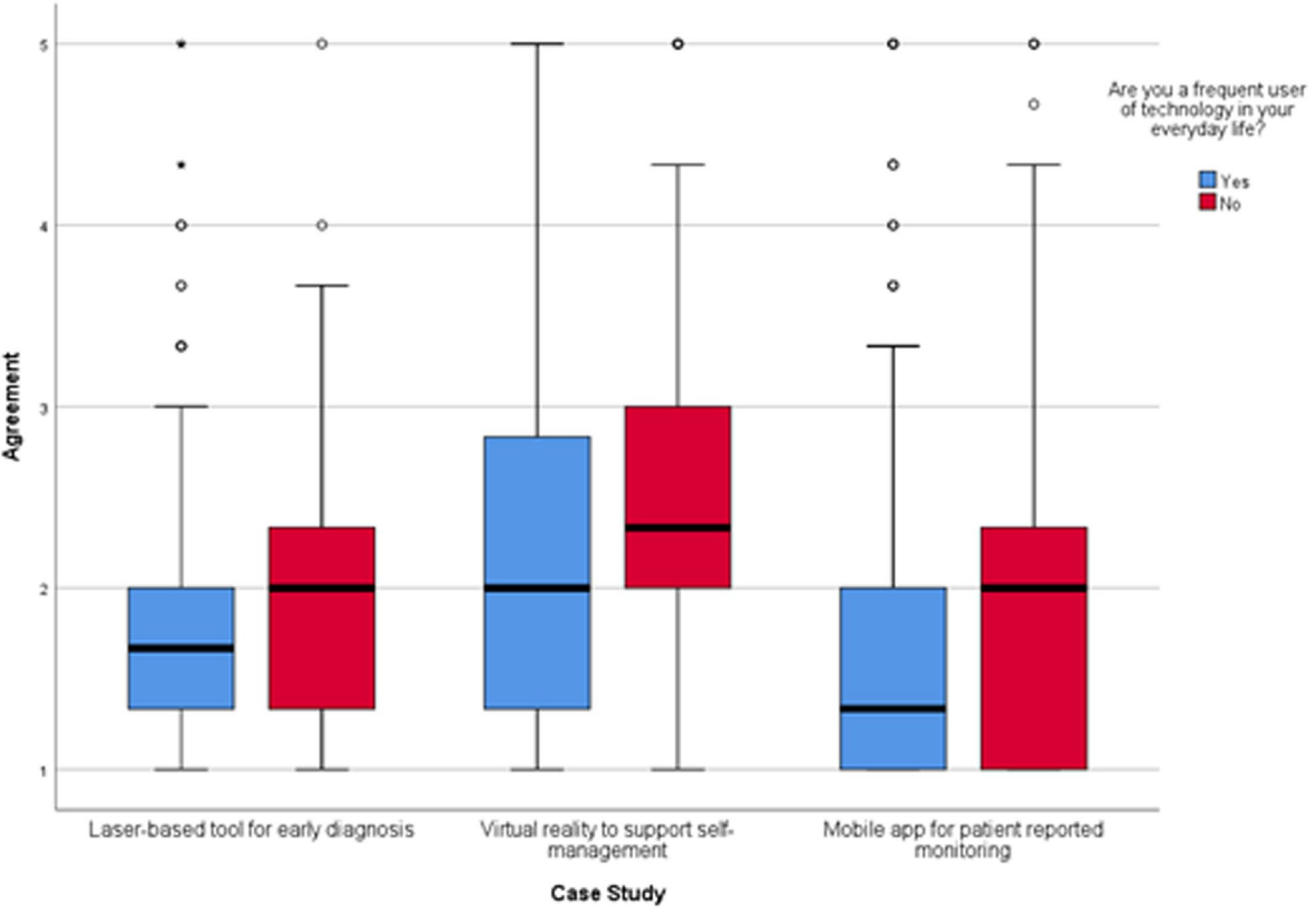
Agreement to accepting laser-based technology, virtual reality and mobile apps in future care, split by respondents who reported being frequent users of technology in everyday life and those who were not frequent users. Agreement score ranges between 1 “Strongly Agree” to 5 “Strongly disagree”. Circles indicate mild outliers (between 1.5 and 3 interquartile ranges away from the 75th percentile) and stars indicate extreme outliers (greater than 3 interquartile ranges away from the 75th percentile). Laser-based tool for early diagnosis n = 1139. Virtual reality to support self-management n = 1089. Mobile app for patient reported monitoring n = 1093

**Fig. 6:**
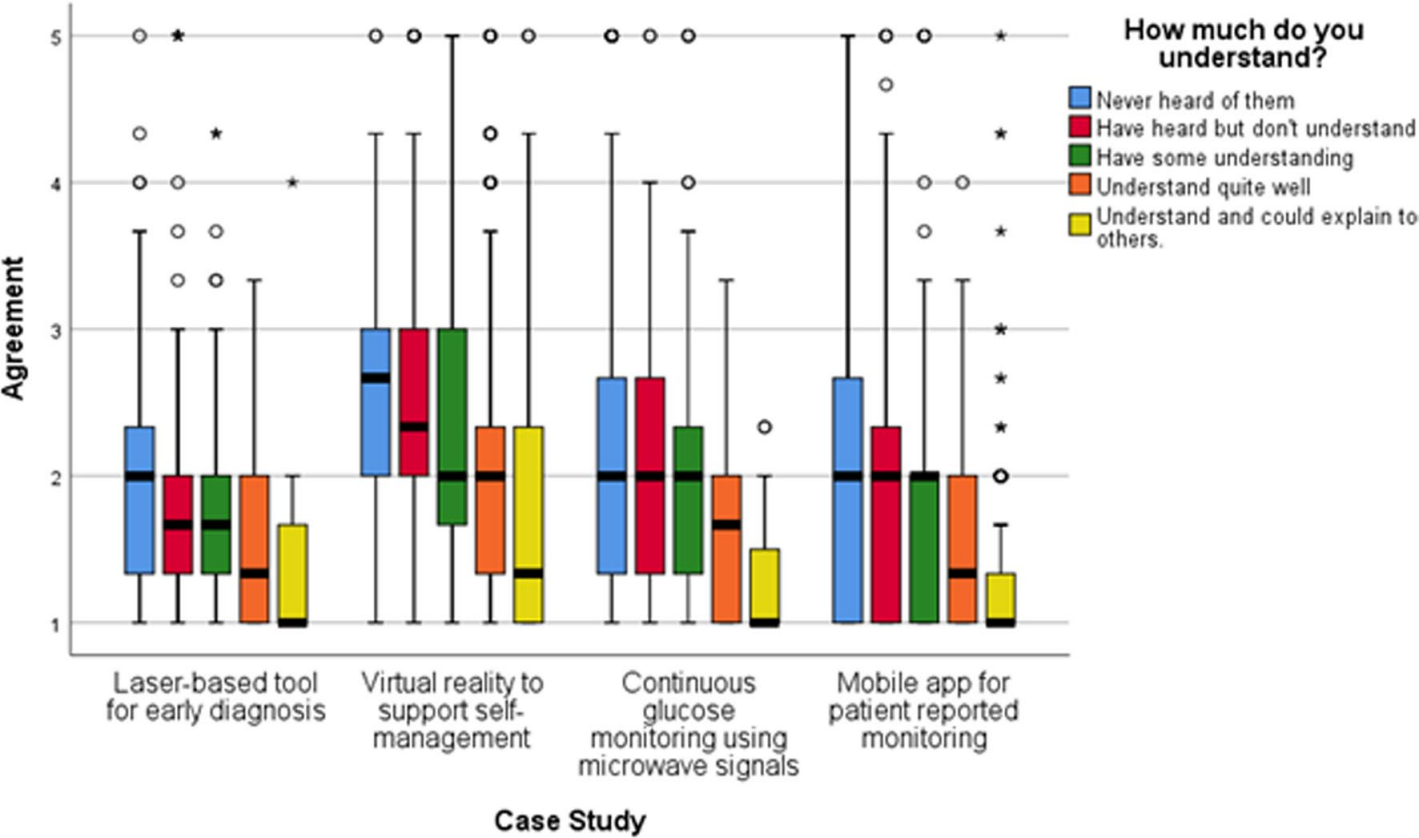
Agreement to accepting NHTs in future care according to respondents’ perceived understanding of how the technology works. Agreement score ranges between 1 “Strongly agree” to 5 “Strongly disagree”. Circles indicate mild outliers (between 1.5 and 3 interquartile ranges away from the 75th percentile) and stars indicate extreme outliers (greater than 3 interquartile ranges away from the 75th percentile). Laser-based tool for early diagnosis n = 1139, Virtual reality to support self-management n = 1089, Continuous glucose monitoring using microwave signals n = 1119, Mobile app for patient reported monitoring n = 1093

As shown in Fig. 5, being a frequent user of technology in everyday life impacted acceptability of all NHTs except for continuous glucose monitoring using microwave signals. Respondents’ self-reported understanding on how the various NHTs work was also linked to acceptability. Across all NHTs, those responders with a self-reported higher baseline understanding (i.e., “Understand and could explain to others”) about how the technology worked were more likely to “Strongly agree” to their use when compared to other responders. See Fig. 6.

No patterns were observed in acceptability in relation to ethnicity, IMD quintile, education, self-reported general health, and self-reported experience with NHTs for health management (Additional file 3).

### Reluctance to accept

When querying reluctance to accept the NHTs after providing information, risks and benefits of each NHT and its proposed application, differences were found between case studies in the reasons for expressing hesitation (Table 2). Between 10% (virtual reality) and 27% (mobile app) of respondents did not know why they were unwilling to accept the technology after reading the information provided. Overall, participants wanted information about the NHTs, and expressed concerns regarding the safety and privacy of using the NHTs, before they would be willing to accept them as part of their care.

**Table 2:** Reasons (frequency of mention) for not accepting the NHTs as part of their care

Item	Category	Laser-based tool for early diagnosis		Virtual reality to support self-management		Continuous glucose monitoring using microwave signals		Mobile app for patient reported monitoring	
		n	%	n	%	n	%	n	%
If you would still not use the technology, why not?	Don't know	62	18	58	10	87	15	70	27
	Provided a reason								
	Safety	**155**	**45**	151	25	**308**	**55**	14	5
	Information	104	30	**186**	**32**	136	24	61	23
	Privacy	3	1	28	5	6	1	**76**	**29**
	Other	7	2	77	13	2	0	14	5
	Test Technology	0	0	45	8	20	4	1	0
	Cost	12	3	25	4	3	1	3	1
	Access	0	0	4	1	0	0	24	9

Bold denotes most frequent reason provided

Analysis to open-ended response options provided additional insight into factors contributing to reluctance to accept the technologies.For the laser-based technology, the most common concern was the risk attached to undergoing surgery. Respondents questioned whether *“benefits would outweigh the risks”* (P622), the *“risk associated when not used properly”* (P1400), and *“risk of localised damage”* (P1435). Respondents therefore also requested more detailed information on the procedure, effectiveness, and risks involved.

The most frequent concern expressed by participants regarding the use of virtual reality to support self-management was the need of additional information about the technology and how it worked. Some respondents struggled to see the benefits of using the NHT, or the added value to more traditional approaches to diabetes management, *“I don't understand what is involved, the time commitment, why it is better than me just eating less and exercising more or the evidence of efficacy”* (P764). Respondents expressed wanting more evidence regarding its effectiveness and implementation. Concerns were also raised about safety, with participants being weary of the possibility of feeling “nauseous”, “sick”, “dizzy” and “disorientated” when using virtual reality. Consequently, the opportunity to test this technology played a role in the decision-making around acceptability: “*I would base my final decision on the results of a demonstration and further information”* (P308).

Similarly, reluctance to accept continuous glucose monitoring using microwave signals was primarily attributable to perceived safety and the long-term effects. Participants expressed particular concern about the impact of microwaves on health. However, respondents also demonstrated that evidence would be an important factor in their decision-making:* “I would like more research done first to show me it’s safe”* (P518).

In the use of mobile apps for patient reported monitoring, concerns about privacy were the most frequently reported reason for non-acceptance, which were not present with the other NHTs. Respondents expressed *“reservations about data security”* (P207), *“privacy concerns over who would have access to the information”* (P881) and *“using of personal information without acknowledgement”* (P1386). Again, respondents indicated that more information would need to be provided before they would accept this NHT (%): *“I would use it if someone took time to explain it fully to me”* (P291).

### Approaches to introduce NHTs to patients

Across all NHTs, most participants (range 53.6% to 65%) preferred receiving information about the NHT verbally, with respondents wanting the information to come from a healthcare specalist or a general practitioner (see Table 3). The second most preferred form of receiving information was via a webpage across all NHTs except for the laser-based technology, where providing information via a visual demonstration was selected to a similar degree as having a verbal conversation about the NHT.

**Table 3:** Method and ideal person to introduce a NHT to future patients

Item	Category	Laser-based tool for early diagnosis		Laser-based tool for early diagnosis		Virtual reality to support self-management		Continuous glucose monitoring using microwave signals	
		n	%	n	%	n	%	n	%
How would you like to receive information? (Can select multiple options)	Verbal conversation	**845**	**58.3**	**777**	**53.6**	**942**	**65.0**	**868**	**59.9**
	Website	640	44.1	591	40.8	631	43.5	705	48.6
	Visual demonstration	505	34.8	725	50.0	606	41.8	597	41.2
	Leaflet	518	35.7	421	29.0	492	33.9	470	32.4
	Other	28	1.9	31	2.1	23	1.6	25	1.7
Who should first tell you about this healthcare technology?	Health professional	**791**	**54.6**	**723**	**49.9**	**551**	**38.0**	**564**	**38.9**
	General practitioner	482	33.2	482	33.2	535	36.9	558	38.5
	Healthcare specialist	81	5.6	71	4.9	177	12.2	126	8.7
	Other	70	4.8	120	8.3	65	4.5	107	7.4
	Family/Friend	55	3.8	38	2.6	56	3.9	77	5.3
	Don’t know	47	3.2	65	4.5	52	3.6	44	3.0
	Nurse	0	0.0	0	0.0	102	7.0	86	5.9
	N/A	32	2.2	31	2.1	55	3.8	63	4.3
	Website	20	1.4	21	1.4	8	0.6	25	1.7
	Pharmacist	0	0.0	0	0.0	2	0.1	6	0.4
	Researcher	0	0.0	0	0.0	2	0.1	0	0.0
	Physiotherapist	0	0.0	0	0.0	0	0.0	1	0.1

Bold denotes most frequent reason provided

## Discussion

Acceptance of an innovative technology is contingent on the attitudes of those who will be using the technology [30, 31]. This survey accrued the public’s attitudes towards NHTs in development; a laser-based tool for early diagnosis, a virtual reality tool to support self-management, a non-invasive continuous glucose monitor using microwave signals, a mobile app for patient reported monitoring. Responses showed that self-reported knowledge about NHTs is diverse, with baseline awareness being highest for mobile apps, followed by laser-based technology, microwave technology and lastly virtual reality. Acceptability of future use varied across the four NHTs, with the highest median acceptability scores achieved for the mobile app and the laser-based technology. Respondents’ self-reported understanding of how the various NHTs worked increased acceptability across all technologies, and frequent users of novel technologies also displayed greater acceptability in all NHTs except for the microwave-based technology. Hesitation about adopting the NHTs was expressed by some participants. Key concerns related to the risks associated with the NHTs and safety. For those respondents who remained hesitant about adopting the NHTs, more information was needed to help inform their decisions, with scientific and clinical evidence the most commonly requested evidence.

Aligned with the literature [20], respondents who reported greater understanding of how a particular NHT works also provided higher acceptance scores to that NHT. Similarly, respondents were more familiar with the healthcare application of laser-based technology and mobile apps, which were also the NHTs that received the highest acceptance scores. These findings parallel research conducted with HCPs showing that perceived lack of technical competency and unfamiliarity generates reluctance to try novel digital solutions [13], and providing ‘how-to’ knowledge (provision of information and training on how to use a new technology properly) at early stages of innovation should be reinforced to enable implementation success [32]. The application of knowledge transfer strategies can empower individuals to make a more informed decision regarding adoption of NHTs.

In contrast to previous work on digital health no trends were found in acceptance scores when considering respondents’ ethnicity, socioeconomic status or level of education. Acceptability scores were higher for female respondents and those who reported frequent use of technology only in some case studies. Further, there was greater acceptability for the mobile app and laser-based technology among the older respondents, who are typically associated with adversity to technology [33]. These findings suggest that we cannot make assumptions about prospective acceptability of a premature NHT based on evidence of similar, existing solutions. The discrepancy between results obtained in this study and previous research may reflect the importance of perceived usefulness and ‘felt need’ on older adults’ valuation of NHTs [33–35]; arthritis is generally an age-related disease for which there is no cure but the proposed NHTs can help slow down its progression through timely action. Though older adults may be more averse to technological solutions, the elevated perceived threat of arthritis may make them more likely to accept NHTs that directly target this condition.

When endorsing a NHT to a patient, information about the NHT should match the preconceptions and knowledge the individual might have of the technology. Safety was an important concern regarding both the laser-based and microwave-based technologies. However, while for laser-based technology participants wanted more information on the surgical procedure and its effectiveness, for the latter there was a call for more evidence on the wider effects of microwaves on human health. This may reflect the high prevalence of incorrect beliefs found on the causal impact of non-ionizing electromagnetic frequencies on cancer risk [36]; misconceptions about microwave signals could have impacted acceptability of its use for glucose monitoring as found in this study. The survey also showed that privacy concerns contribute to reluctance on accepting a novel intervention using a mobile app, but not one relying on virtual reality, which is also vulnerable to disclosure of personal data [37]. The literature on these technologies applied to other health conditions shows a similar trend (see 33,34). Information provided about an NHT needs to be tailored to address existing preconceptions, rather than a brief outline as provided in this study.

It is noteworthy that at face value findings from this survey appear to align with the ‘Deficit Model’, which postulates public scepticism towards science arises from a dearth of information. However, examination of trends across NHTs suggests that this view is over-simplistic and adds to the extensive critique of this approach to conceptualising public attitudes (e.g., 40). Attitudes towards NHTs appear to vary between population groups and according to the values of each individual (e.g., balancing the risk of innovation with the relevance of the therapeutic area to the individual). Further, the myriad of responses provided indicate that for information to be effective in changing attitudes towards NHTs, the information provided needs to be tailored to the needs and preferences of each individual.

A considerable strength of this study is the use of a large-scale survey to obtain insight on attitudes towards innovative technological solutions that are still at early stages of development. Both the people designing and developing these NHTs (including authors of this manuscript) and implementation scientists can use results to prospectively mitigate obstacles to acceptance and adoption. For example, respondents who struggled to understand how virtual reality works also showed greater reluctance to accept the technology when presented with its possible application to support diabetes self-management. Results from this survey suggest that enabling target beneficiaries to trial the technology could increase acceptability. The value of inserting a ‘trial phase’ when implementing this technology aligns with evidence that older adults change their attitudes towards virtual reality after using it [38]. Further, the survey evaluated four very different NHTs that target different healthcare challenges, enabling comparability between the technological solutions and highlighting the importance of NHT decision-making to be conducted on a case-by-case basis (i.e., each technology and its application).

There are, however, certain limitations that need to be considered. The technological solutions presented in this study varied across different factors (e.g., medical application, function), but the majority focussed on disease management. It might be worthwhile to explore trends in attitudes towards NHTs designed for a broader range of facets of healthcare provision (e.g., diagnostic vs. general management vs. preventative). This study was based on an online survey, which means that the attitudes of people who do not normally use the internet were not captured. The majority of respondents did not report a diagnosis of the medical conditions presented, which is likely to influence how likely individuals would accept a technology if they were experiencing the adverse symptoms of a condition. The survey also explored attitudes and prospective acceptability of NHTs. Though acceptance is closely linked to behavioural intention of using technology [31], it cannot account for experienced usability [30, 41]. Although the inclusion of open-ended questions in the survey provided some insight into factors contributing to reluctance to accept NHTs, more in-depth qualitative methodologies (e.g., interviews and focus groups) would provide a more comprehensive and nuanced understanding. Further research is also needed to evaluate how the outcomes from this survey reflect future adoption of the NHTs when implemented.

### Practical implications

This study emphasises the complex task stakeholders face in the development and implementation of NHTs, where it is impossible to predict attitudes to future NHTs on the basis of existing solutions. Insight into the public’s attitudes towards proposed NHTs can guide product development and early identification of implementation strategies [12, 42]. Understanding public attitudes towards proposed technological solutions can assist funders and regulatory agencies in their decision-making, and the preparation of blueprints for introducing NHTs in routine practice that can adapt to the type of technology, its application and the users of the technology. It can also assist researchers ensure that innovation is inclusive, and that sustainable support is in place to assist future beneficiaries access the healthcare solution [13, 43]. Additionally, the relevance of an individual’s understanding of the NHTs and preference for direct conversations with health professionals strongly supports the adoption of shared decision-making practices [44] to increase acceptance of NHTs in health and social care. The request for more information to guide decision-making around acceptability highlights the importance of developing regulatory frameworks to build the evidence base on safety and effectiveness of NHTs and sharing it with patients during clinical decision-making.

## Conclusion

This study examined UK public attitudes and opinions towards NHTs being used to address common chronic conditions. Responses suggest that the UK public is generally open to technological innovation in their future care but acceptability of a specific NHT is likely to vary. When considering factors that might explain such variation (e.g., age, gender, familiarity with technology), this study demonstrated the need to avoid generalisations across technologies; the relevance of individual/group characteristics on acceptability differed across case studies. Similarly, reluctance to accept the NHTs was driven by differing rationales, which might be linked to an individual’s understanding of the technology, or the perceived balance of benefits versus costs of using the NHT as a targeted solution for a specific clinical need. By adopting a research framework that encompasses several NHTs in development, this study evidences the complexity underpinning acceptability research in the field of healthcare technology innovation. A comprehensive evaluation of how future beneficiaries might respond to a technological solution as it is being conceptualised has the potential to streamline development and implementation and maximise its health and societal impact.

## Supplementary Information

**Additional file 1.** Survey and case studies presented on the novel healthcare technologies selected.

**Additional file 2.** Demographic characteristics of respondents.

**Additional file 3.** Acceptance of NHTs in future care according to respondents’ individual characteristics.

## Data Availability

The datasets used and analysed during the current study are available from the corresponding author on reasonable request.

## Abbreviations

NHT: Novel healthcare technology
HCP: Healthcare professional
IMD: Index of Multiple Deprivation
